# Supplementary material for: T-cell trans-synaptic vesicles are distinct and carry greater effector content than constitutive extracellular vesicles
Source: Nat Commun. 2022 Jun 16;13:3460. doi: 10.1038/s41467-022-31160-3 (PMC9203538; doi:10.1038/s41467-022-31160-3)
Supplement: Supplementary file 1 — Supplementary Information [file 41467_2022_31160_MOESM1_ESM.pdf]

SUPPLEMENTARY INFORMATION

T cell trans-synaptic vesicles are distinct and carry greater effector content than constitutive extracellular vesicles.

Pablo F. Céspedes, Ashwin Jainarayanan, Lola Fernández-Messina, Salvatore Valvo, David G. Saliba, Elke Kurz, Audun Kvalvaag, Lina Chen, Charity Ganskow, Huw Colin-York, Marco Fritzsche, Yanchun Peng, Tao Dong, Errin Johnson, Jesús A. Siller-Farfán, Omer Dushek, Erdinc Sezgin, Ben Peacock, Alice Law, Dimitri Aubert, Simon Engledow, Moustafa Attar, Svenja Hester, Roman Fischer, Francisco Sánchez-Madrid, Michael L. Dustin.

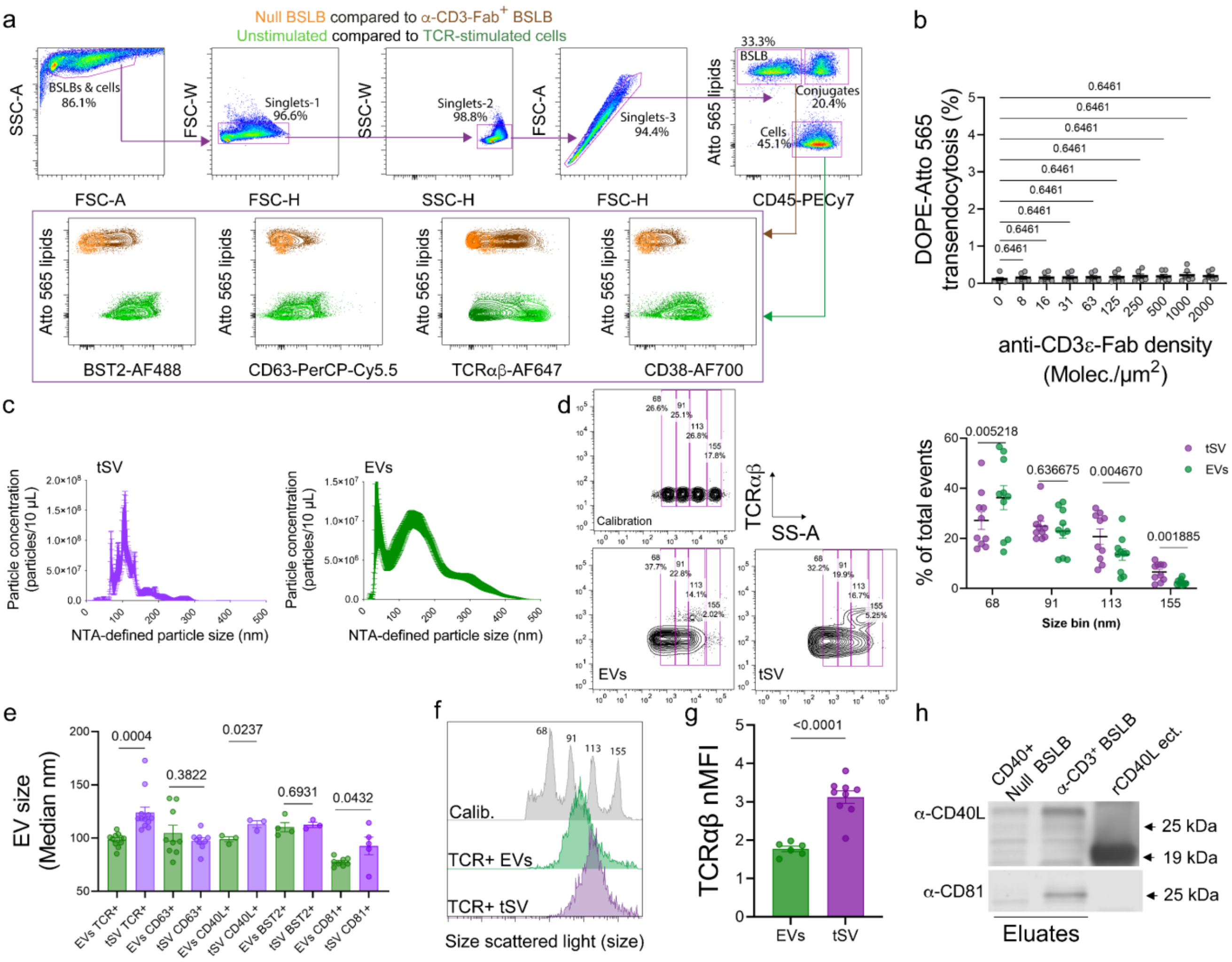

Supplementary Figure 1. **Multiparametric analyses of released tSV show a highly heterogeneous population of vesicles being transferred at the IS. a** T cell: BSLB synapses and the release of synaptic ectosomes containing engaged T cell molecules can be studied by conventional multicolor flow cytometry. Shown is the representative gating strategy to discriminate single BSLB and T cells after conjugate dissociation (as in Figure 1b). Shown are also the biparametric overlays of single BSLBs either null (orange), agonistic (brown) and non-stimulated (light green) and TCR-stimulated cells (dark green) for BST2, CD63, TCRαβ and CD38. This gating strategy was also used for identifying single cells and BSLBs in Fig. 2, Fig.3, Fig. 4 and Fig. 5. **b** Conventional FCM analyses of T cells after conjugate dissociation shows negligible uptake of fluorescent lipids by the stimulated T cells, which is observed across a broad range of α-CD3ε Fab densities. Data shown represent the % of the total Atto 565 signal observed in null BSLB. **c** Nanoparticle Tracking analysis size distribution of tSV and EVs isolated by differential centrifugation. **d** Using size calibration beads (top panel), EVs (green and bottom left panel) and tSV (violet and bottom right panel) were compared across different bin sizes (SS-A range) as defined by the coefficient of variation (CV) of the standard bead population. **e** Size distribution measured by NanoFCM of TCR, CD63, CD40L, BST2 and CD81 positive EVs (green) and tSV (violet). **f** Example overlaid histograms showing the comparison of TCRαβ^{+} vesicle size in EVs (green) and tSV (violet) using benchmark calibration silica beads as reference (grey). **g** NanoFCM for the comparison of TCRαβ expression in EVs (green) and eluted tSV (violet). Expression was normalized to the signal measured in isotype-labelled controls (TCRαβ MFI / Isotype MFI). **h** Immunoblot

showing the size comparison between tSV-associated CD40L and a recombinant human CD40L ectodomain. Null BSLB were loaded only with ICAM1 and CD40. Data represent Means ± SEM from n = 4 independent experiments, each dot in (b), (d-e) and (g) represent biologically independent samples. Normality was determined using Shapiro-Wilk test and statistical significance was determined by One-Way ANOVA (b) and Multiple paired t-test (d) corrected by two-stage step-up method of Benjamini, Krieger and Yekutieli (FDR Q= 0.05), and by unpaired two-tailed t-test (e, g) to compare tSV and EVs per independently per marker. Values on top of scatter plots represent adjusted P values. Uncropped and unprocessed versions of immunoblots shown in (h) are available as source data.

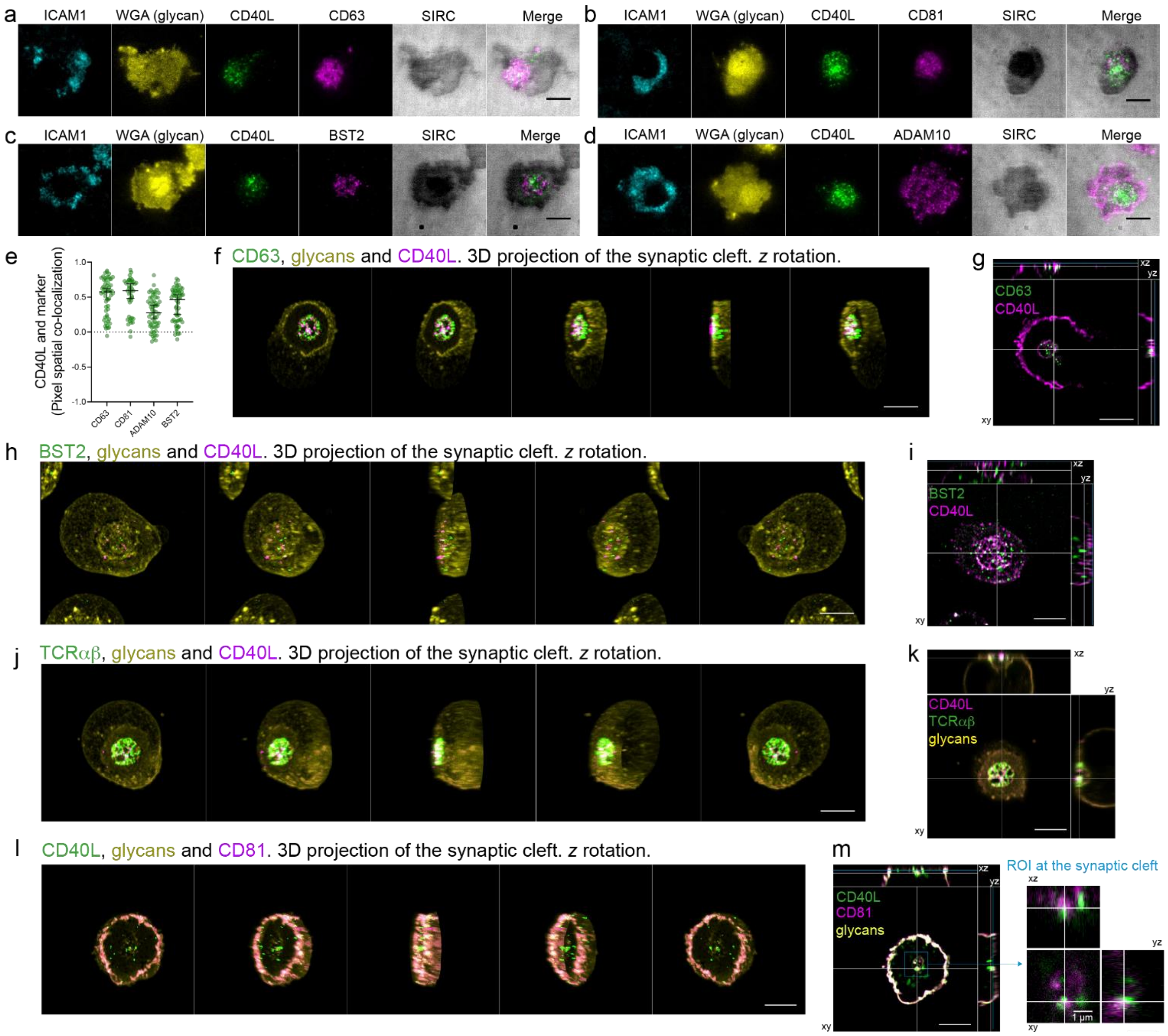

Supplementary Figure 2. **TIRFM and Airyscan confocal imaging of T cell synapses indicate a high spatial segregation of T cell tSV. a-d** Representative TIRFM microphotographs showing the discrete spatial distribution of CD40L and other extracellular vesicle markers in non-permeabilized and surface-stained TH synapses. The synaptic distribution of CD40L and other tSV markers, including CD63 (**a**); CD81 (**b**); BST2 (**c**); and ADAM10 (**d**, as control) is shown. **e** Pearson's correlation coefficient (PCC) for the spatial colocalization between CD40L and other tSV markers. Each dot represents the average PCC per independent synapse with representative TIRFM images shown in **a-d**. **f-i** Airyscan microphotographs of surface stained TH immune synapses showing the 3D spatial distribution of CD40L and CD63 (**f, g**) BST2, (**h, i**) TCRαβ (**j, k**), and CD81 (**l, m**) on synapses formed between TH and SLB after 15 min of interaction. Glycans in the plasma membrane and tSV are labelled with Wheat Germ Agglutinin CF594 (WGA). SIRC: surface interference reflection contrast microscopy. Scale bar = 5 µm. **g, i, k** and **m** show orthogonal views of TH synapses with the *z*-plane crossing the synaptic cleft. Scale bar = 5 µm. In **a-m** SLB contained 200 molec./μm^2 of ICAM1, 20 molec./μm^2 of CD40 and 30 molec./μm^2 of α-CD3ε Fab. Data representative of 2 independent experiments with n = 2 biologically independent samples (donors) (a-m).

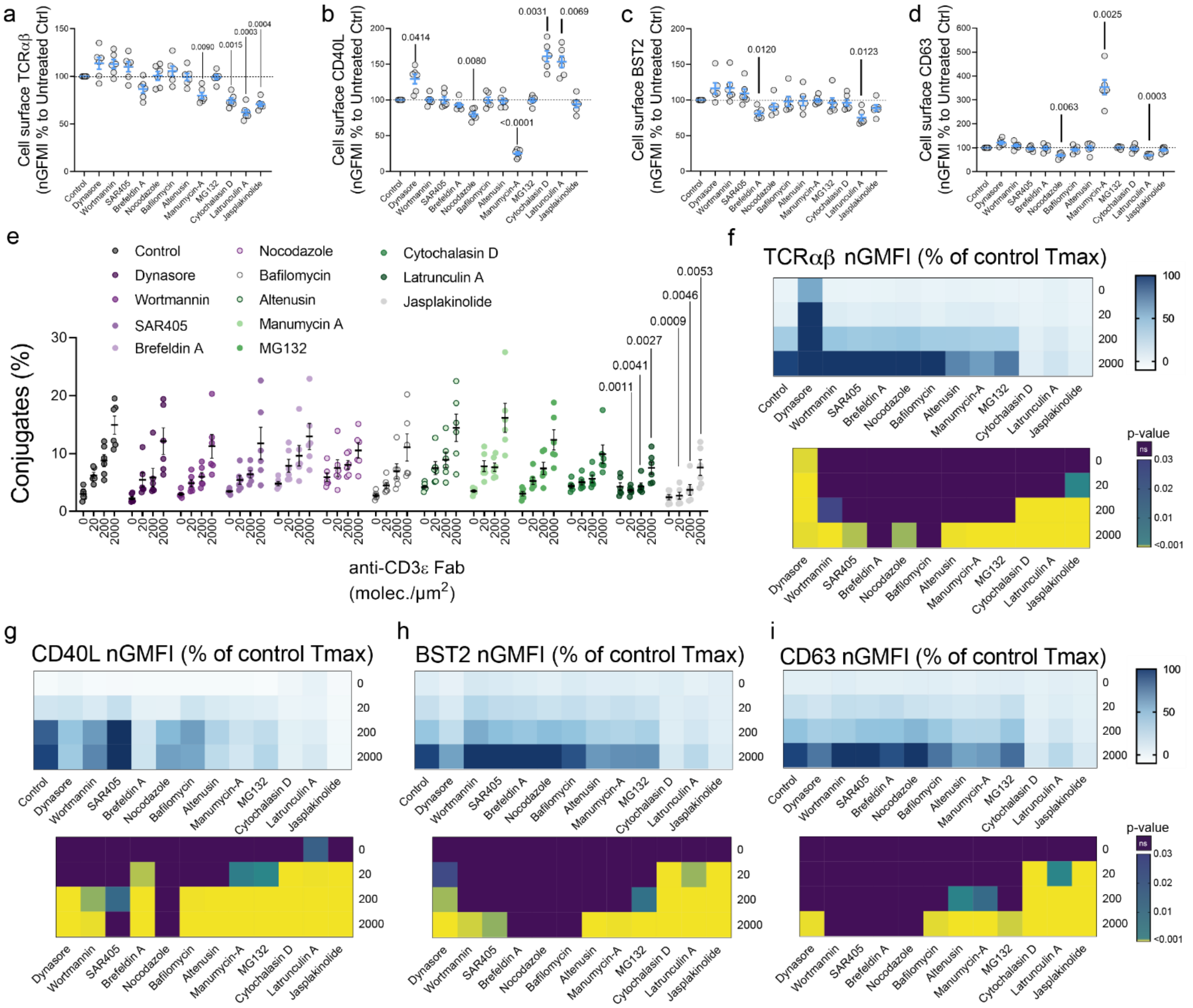

Supplementary Figure 3**. The output from different subcellular compartments underpins tSV heterogeneity**. TH were preincubated for 30 min with a panel of 12 inhibitors and then co-cultured with control and agonist BSLB at a 1:1 ratio for another 90 min while keeping inhibitor concentrations (see Supplementary Data 1). **a-d** Cell surface expression of TCRαβ (**a**), CD40L (**b**), BST2 (**c**) and CD63 (**d**) after inhibitor treatment. **e** The stability of cellular contacts was assessed by tracking the percent of remaining cell: BSLB conjugates after cold incubation. **f-i** Transfer of TCRαβ$^+$ (**f**), CD40L$^+$ (**g**), BST2$^+$ (**h**), and CD63$^+$ (**i**) tSV to BSLB compared and normalized to the maximum transfer (Tmax) observed per donor in control untreated cells (at 2,000 molec./μm^2). A representative gating strategy to identify single BSLBs and single cells is shown in Supplementary Fig. 1a. Data represent means ± SEM for n = 6 biologically independent samples (donors) analyzed across two independent experiments. Values on top of scatter plots represent adjusted P values. Bottom heatmaps display adjusted P values for the comparison of tSV transfer between the control and different inhibitors, and across different α-CD3ε-Fab densities. Statistical significance and P-values were determined using One-way ANOVA and Dunnett's correction for multiple comparisons (a-d), and two-tailed multiple t-test corrected for multiple comparisons by either two-stage step-up method of Benjamini, Krieger and Yekutieli (FDR, Q = 0.01)(e), or Holm-Šídák test (α = 0.05) for the

multiple comparisons (f-i) across different α-CD3ε Fab densities. Data from n = 6 biologically independent samples (donors) across two independent experiments. BSLB presenting 200 molec./μm^2 ICAM1, 20 molec./μm^2 of CD40 and increasing densities of anti-CD3ε Fab were used in all experiments.

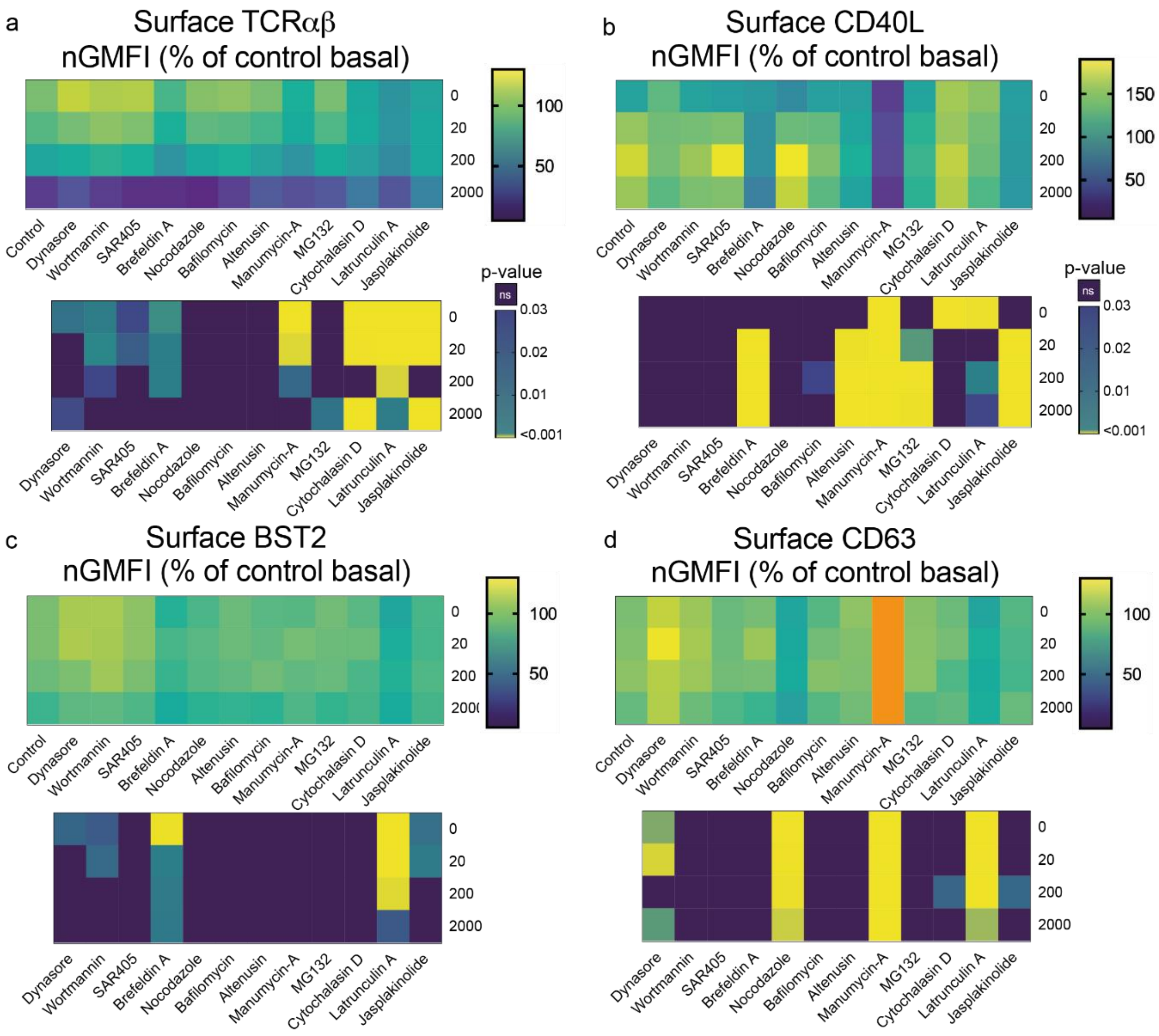

Supplementary Figure 4. **Surface expression of tSV markers following inhibitor treatment. a-d** Dynamics of TCRαβ (**a**), CD40L (**b**) BST2 (**c**) and CD63 (**d**) cell surface expression normalized to the baseline of untreated controls. Right heatmaps show *P*-values for the comparison of surface marker expression between the controls and different inhibitors, and across different α-CD3ε-Fab densities. In **d** out of range values (>200%) are represented in orange. Statistical significance and P values were determined using two-tailed multiple t-test with Holm-Šídák test (α = 0.05) correction for the multiple comparisons of marker cell surface expression across different α-CD3ε Fab densities and among inhibitor treated cells and controls (a-d). A representative gating strategy to identify single BSLBs and single cells is shown in Supplementary Fig. 1a. Data represent means from n = 6 biologically independent samples (donors) across two independent experiments. BSLB presenting 200 molec./μm^2 ICAM1, 20 molec./μm^2 of CD40 and increasing densities of anti-CD3ε Fab were used in all experiments.

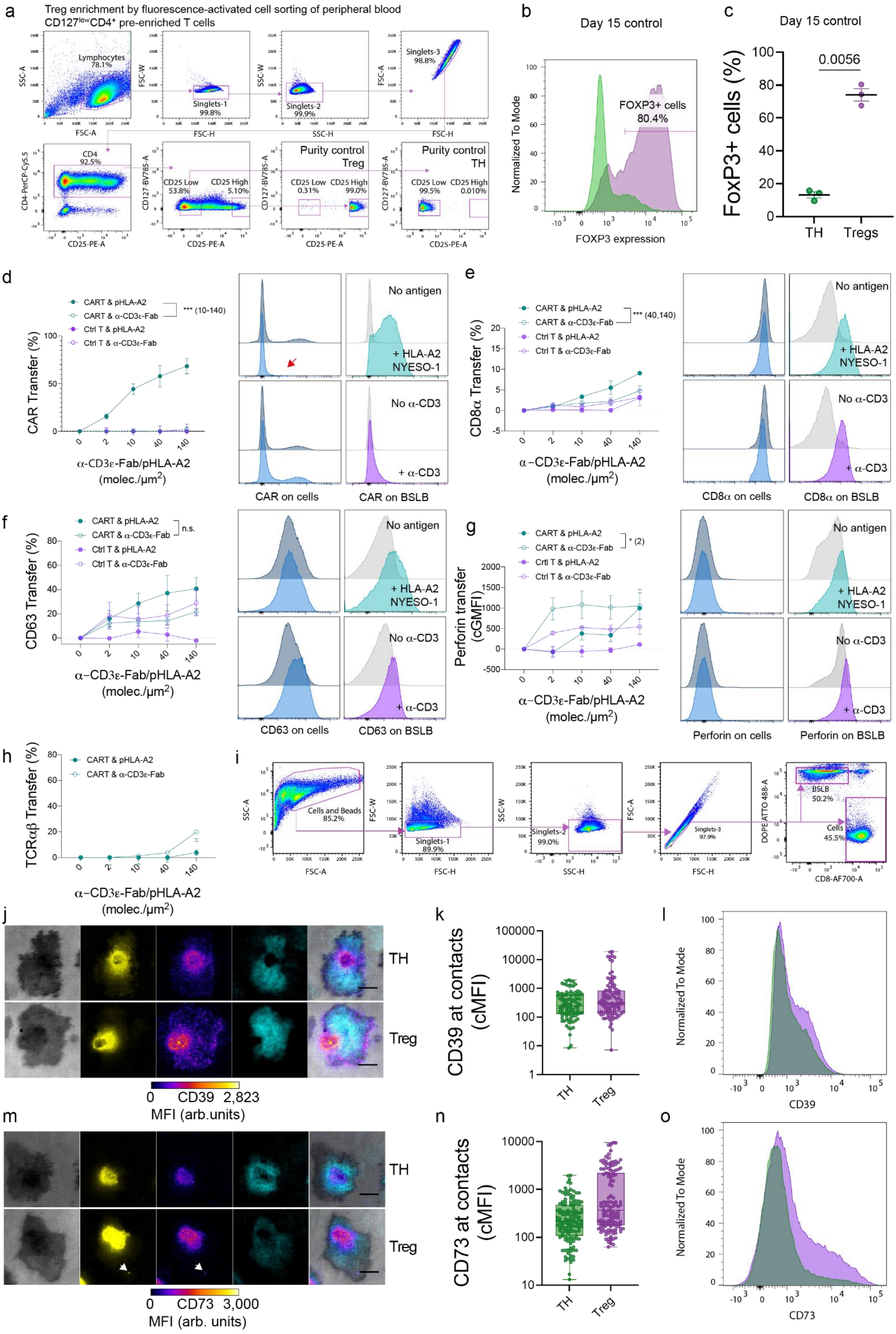

a
Treg enrichment by fluorescence-activated cell sorting of peripheral blood
CD127lowCD4+ pre-enriched T cells
Lymphocytes 78.1%
Singlets-1 99.8%
Singlets-2 99.9%
Singlets-3 98.8%
CD4 92.5%
CD25 Low 53.8%
CD25 High 5.10%
Purity control Treg
CD25 Low 0.31%
CD25 High 99.0%
Purity control TH
CD25 Low 99.5%
CD25 High 0.010%
b
Day 15 control
FOXP3+ cells 80.4%
Normalized To Mode
FOXP3 expression
c
Day 15 control
0.0056
FoxP3+ cells (%)
TH
Tregs
d
CART & pHLA-A2
CART & α-CD3ε-Fab
Ctrl T & pHLA-A2
Ctrl T & α-CD3ε-Fab
*** (10-140)
CAR Transfer (%)
α-CD3ε-Fab/pHLA-A2 (molec./μm2)
No antigen
+ HLA-A2 NYESO-1
No α-CD3
+ α-CD3
CAR on cells
CAR on BSLB
e
*** (40,140)
CD8α Transfer (%)
CD8α on cells
CD8α on BSLB
f
n.s.
CD63 Transfer (%)
CD63 on cells
CD63 on BSLB
g
* (2)
Perforin transfer (cGMFI)
Perforin on cells
Perforin on BSLB
h
TCRαβ Transfer (%)
i
Cells and Beads 85.2%
Singlets-1 89.9%
Singlets-2 99.0%
Singlets-3 97.9%
BSLB 50.2%
Cells 45.5%
j
TH
Treg
0 CD39 2,823
MFI (arb.units)
k
CD39 at contacts (cMFI)
l
CD39
m
TH
Treg
0 CD73 3,000
MFI (arb. units)
n
CD73 at contacts (cMFI)
o
CD73

Supplementary Figure 5**. T cell phenotype influences tSV composition, the exemplary case of Treg and CART cells. a** Gating strategy for the isolation of Treg cells from peripheral blood. Post-sort purity controls are shown on the bottom right panels. The expansion of FoxP3$^+$-enriched CD4^{hi} T cell cultures starts with the isolation of cells from peripheral blood based on CD4 expression and the negative expression of CD127 and of the null (TH) or highest (5%) expression of CD25 (Treg). **b** Example of intracellular staining for FOXP3 on day 15 of expansion of TH (green histograms) and Treg (violet histograms). **c** Frequency of FOXP3$^+$ cells on day 15 of TH (green circles) and Treg (violet circles) expansion. **d-i** BSLBs can also be tailored for the study of CART cell tSV and the transfer of the chimeric antigen receptor (CAR) (**d**), the TCR co-receptor CD8α (**e**), CD63(**f**) and Perforin(**g**). Non-CAR cells (open (α-CD3ε–stimulated) and closed (antigen-stimulated) violet circles) are shown for comparison (n= 2 independent biological samples). **d-g** Representative half-overlaid histograms are shown to the right. The differences in synaptic transfer at comparable α-CD3ε (teal, open circles) and HLA-A2: NY-ESO-1 (teal, closed circles) densities (140 molec./μm^2). TSV transfer to BSLBs instigated by either the CAR antigen (top panels; teal histograms), or the α-CD3ε Fab (bottom panels, violet histograms) are shown for each marker. Cells with no antigen/α-CD3 (dark blue histograms), and stimulated with either HLA-A2: NY-ESO-1 or α-CD3ε (light blue). Light grey histograms represent non-agonistic (null) BSLBs . **h** for comparison, TCRαβ transfer is shown for the tested densities in n = 2 independent CART biological samples. **i** Gating strategy for identifying single BSLBs and single CART shown in d-g. **j-o** Central clustering of CD39 (**j**) and CD73 (**m**) in Treg synapses formed on SLB containing 200 molec./μm^2 of ICAM1, 20 molec./μm^2 of CD40 and 30 molec./μm^2 of α-CD3ε Fab and imaged with TIRFM. MFI = Mean Fluorescence Intensity, arb. units = arbitrary fluorescence units. SIRC: surface interference reflection contrast. Cell surface glycans are labelled with Wheat Germ Agglutinin (WGA). White arrowheads show released WGA$^+$ CD73$^+$ vesicles (diffraction-limited). Scale bar = 5 μm. **k, n** Background corrected MFI for CD39 (**k**) and CD73 (**n**) in TH (green circles) and Treg (violet circles) synapses. **l, o** Ectonucleotidase incorporation in tSV relates to increased surface expression of CD39 (**l**) and CD73 (**o**) in Treg cells (violet histograms) compared to TH (green histograms). Data represent means ± SEM of n = 3 (c), 4 (d-g), and 2 (h-o) independent biological samples across 3 (c) and 2 independent experiments (d-o). Normality was determined using Shapiro-Wilk test and statistical significance was determined by two-tailed unpaired t-test (**c**) and two-tailed Multiple t-test with Holm-Šídák correction (α = 0.05) for the multiple comparisons of synaptic output between CART stimulated with antigen and CART stimulated with anti-CD3ε Fab. Values on top of scatter plots represent adjusted P values. In a, e, and g adjusted P-values are represented as *p≤0.05, **p≤0.005, ***p≤0.0005, ****p≤0.0001 and ns = not significant.

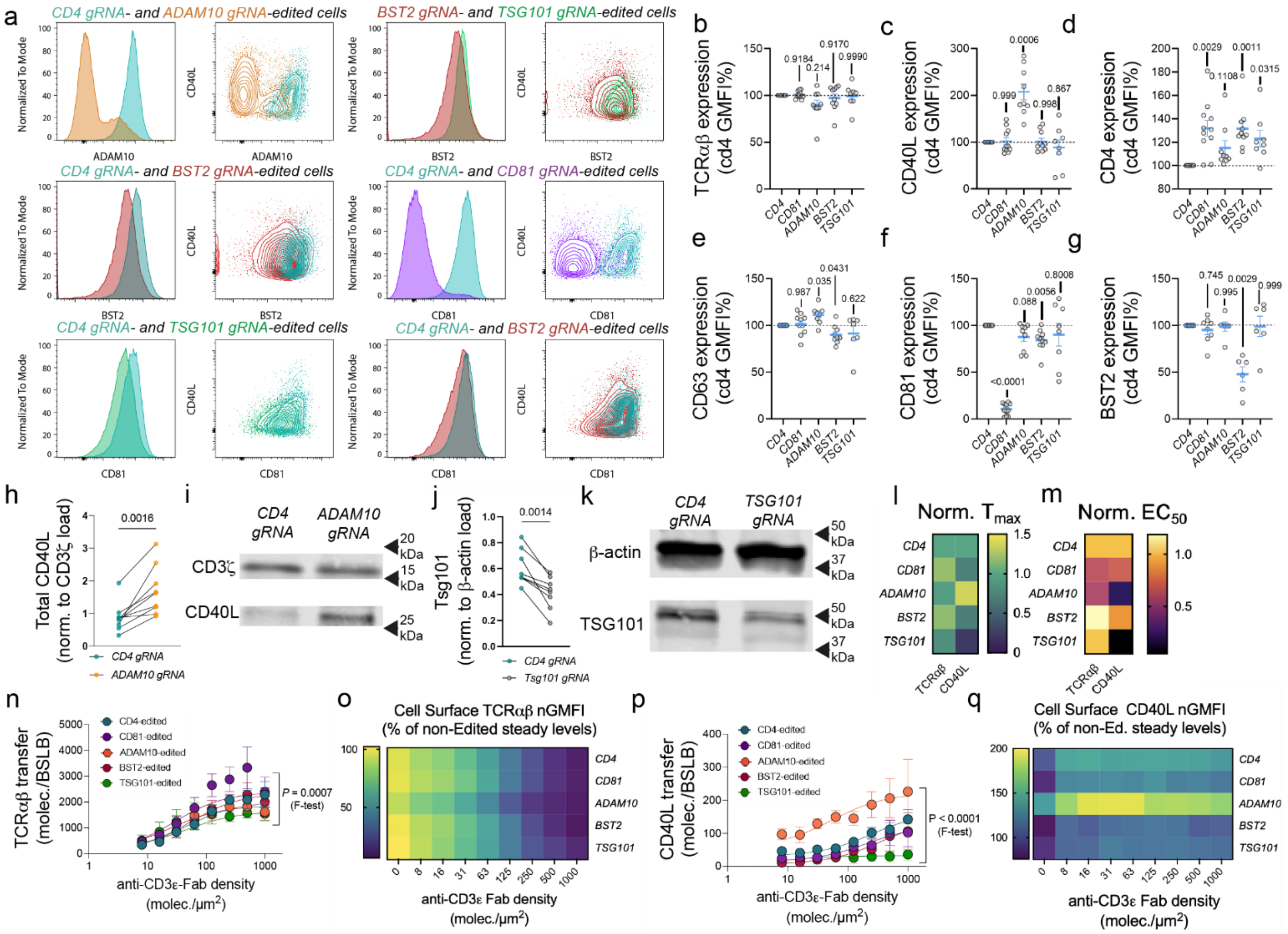

Supplementary Figure 6. **CRISPR/Cas9 controls. a-f** Flow cytometry measurements of cell surface protein expression in TH cells with CD4, CD81, ADAM10, BST2, and TSG101 genes disrupted with CRISPR/Cas9. **a** Representative half-overlaid histograms and bi-parametric contour plots showing the surface expression levels of edited proteins (headings) and their relationship with the surface expression of CD40L (*y* axis). The expression of TCRαβ (**b**), CD40L (**c**), CD4 (**d**), CD63 (**e**), CD81 (**f**) and BST2 (**g**) was normalized to the levels of CD4-edited controls. **h** CD40L expression normalized to CD3ξ expression in *CD4*- (teal circles) and *ADAM10*-edited (orange circles) cells. **i** Representative immunoblots for CD40L and CD3ξ expression in whole cell lysates. **j** TSG101 expression normalized to β-actin in CD4- (teal circles) and TSG101-edited cells (grey circles) . **k** Representative immunoblots showing the downregulation of TSG101. **l-m** Dynamics of TCR and CD40L tSV transfer to BSLBs for different CRISPR/Cas9-edited cells shown as normalized T_{max} (l) and EC_{50} (m). **n** TCRαβ$^+$ tSV (as TCRαβ$^+$ molec./BSLB) release at increasing α-CD3ε Fab densities. **o** Surface TCRαβ expression on CRISPR/Cas9-edited TH cells cocultured with BSLBs as in (n). **p** CD40L$^+$ tSV (as CD40L$^+$ molec./BSLB) release at increasing α-CD3ε Fab densities. **q** Changes in surface CD40L upregulation on CRISPR/Cas9-edited TH cells cocultured with BSLBs as in (p). Representative gating strategy for the identification of single BSLBs and cells is shown in Supplementary Fig. 1a. Data represents Means ± SEM of n = 8 (b-g, h), and 7 (j) biologically independent samples (donors) and experiments. In n-q, data represent means (o, q) ± SEM (n, p) of n = 9 (*CD4*), 7 (*CD81*), 6 (*ADAM10*), 6 (*BST2*) and 3 (*TSG101*) biologically independent samples (donors) and experiments. Normality was determined using Shapiro-Wilk test and statistical significance was determined by One-Way ANOVA with Geisser-Greenhouse correction and mixed effects model corrected for multiple comparisons using

Dunnett's (b-g) and two-tailed paired t-test (h, j). Non-linear regression using three parameters F-test was used to compare the dynamics of CD40L and TCRαβ transfer to BSLBs among different CRISPR/Cas9 gene ablations (l, m, n, p). Uncropped and unprocessed versions of immunoblots shown in (i) and (k) are available as source data.

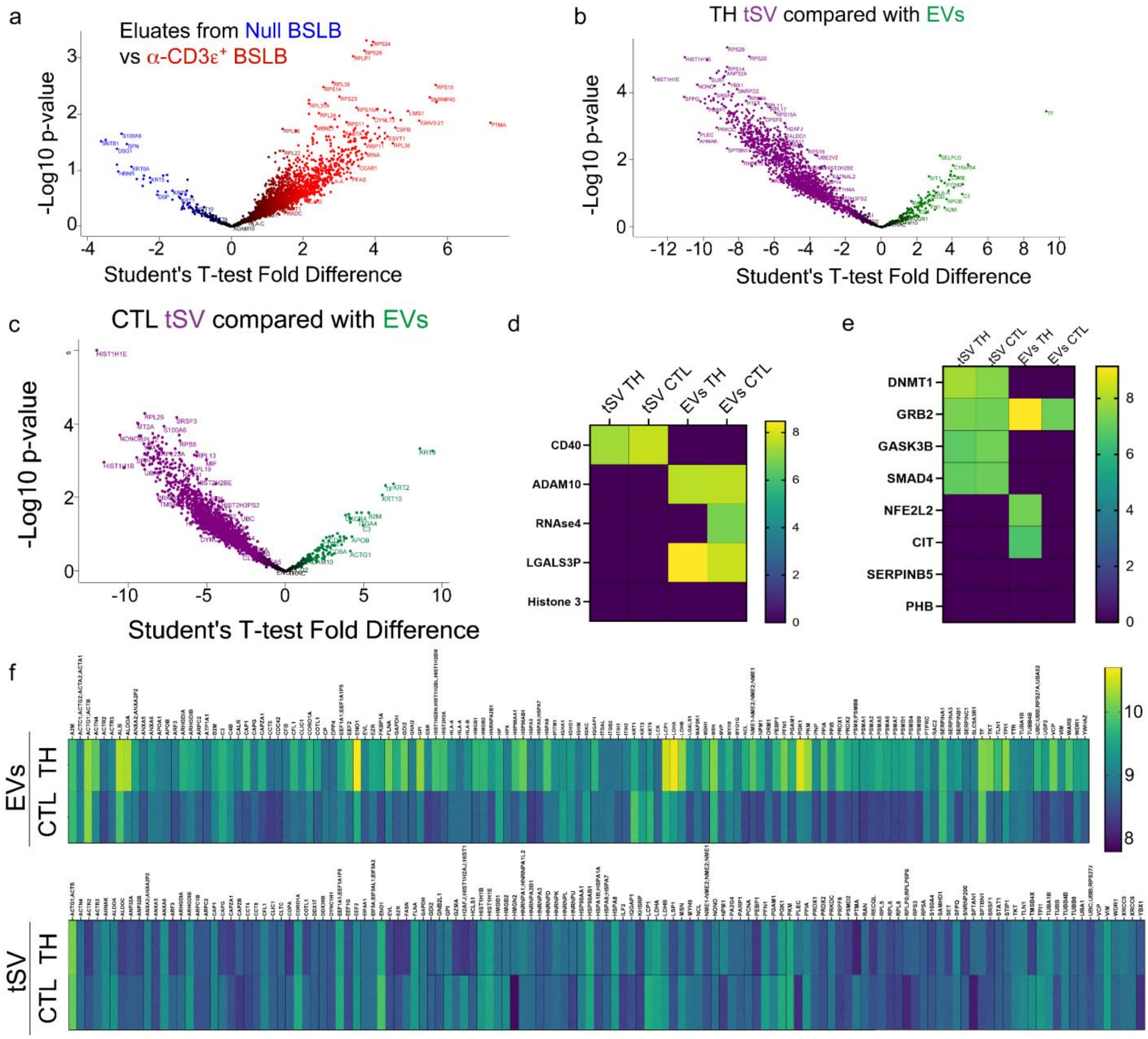

Supplementary Figure 7. **TSVs have distinct protein composition than EVs.** **a** LC-MS/MS analyses of eluted tSVs from α-CD3$^+$ and null BSLB (containing only ICAM1 and CD40). **b** differentially expressed proteins found in **a** tSV were then used for the comparison between tSV and EVs produced by TH. **c** As in (b), comparison between tSV and EVs of CTL. In a-c, p-values were determined by two-tailed student T-test. **d** LC-MS/MS analyses reveal the differential enrichment of contaminating proteins in tSV and EVs. **e** tSV and EVs showing the differential enrichment of protein targets for miR identified in Fig. 6. **f** Enriched proteins in tSV and EVs were identified by averaged-based normalization of peptide intensities (log10). Data represent means from n = 4 biologically independent samples across four independent experiments.

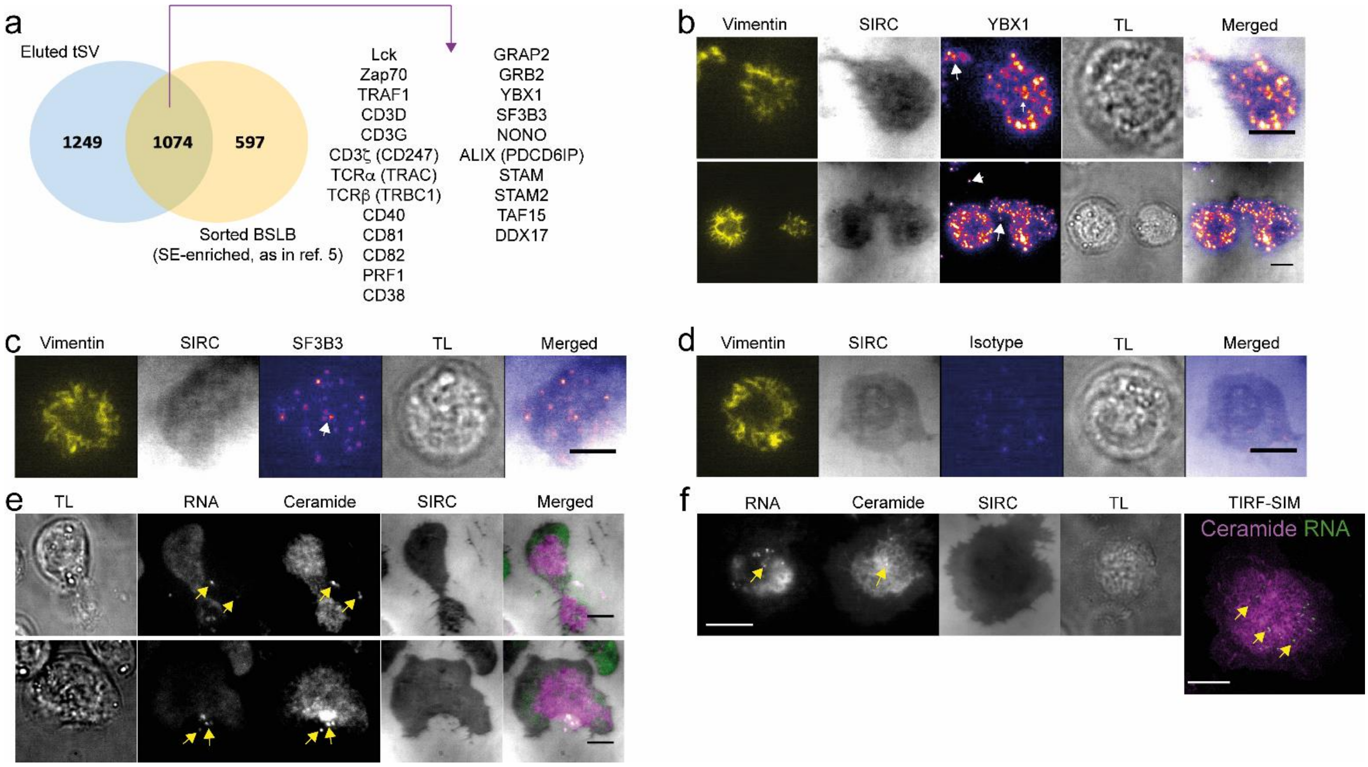

Supplementary Figure 8. **RNA-binding proteins (RBPs) and RNA are mobilized to the synaptic pole of T cells and released as part of tSV. a** Venn-Diagram showing the co-identification of tSV immune-receptors and RBPs in LC-MS/MS datasets generated by two different isolation methods, namely tSV elution (light blue circle) and sorting of BSLB (yellow circle), which are enriched in synaptic ectosomes (SE)[5]. **b** TIRFM imaging of the RNA-binding protein YBX1 in fixed and permeabilized TH synapses formed after 20 minutes of interaction with SLBs. YBX1 was enriched in TH tSV as defined by LC-MS/MS analyses here and in previously published proteomics from isolated SE[5]. Vimentin was used for the identification of the pSMAC (yellow) and the cSMAC (dark central area). White arrows indicate the localisation of YBX1 in vesicular tracks of cells resuming migration and in the synaptic cleft. **c** as in (**b**), T cells were stained against SF3B3. **d** A rabbit monoclonal IgG was used as isotype control for the experiments shown in **b** and **c**. **e-f** TH cells were pre-stained with BODIPY TR Ceramide and SYTO RNASelect to track the localization of ceramide and membrane enriched structures in the synaptic cleft of live cells. **e** Yellow arrows show the release of ceramide$^+$ and RNA$^+$ vesicles in the tracks of TH cells resuming migration. **f** Example of a TH synapse showing localization of ceramide$^+$ and RNA$^+$ puncta in transit to the cSMAC. *Right*: We used the super-resolution provided by eTIRF-SIM to visualize the arrangement of RNA$^+$ puncta at 5 min of synapse assembly. Most puncta associate to ceramide enriched ER-like structures lining the cSMAC (synaptic cleft, yellow arrows), suggesting the rapid polarization of RNA transport machineries to the boundary of the synaptic cleft. Scale bars = 5 µm. Imaging representative of at least three independent experiments and n = 4 biologically independent samples (donors).

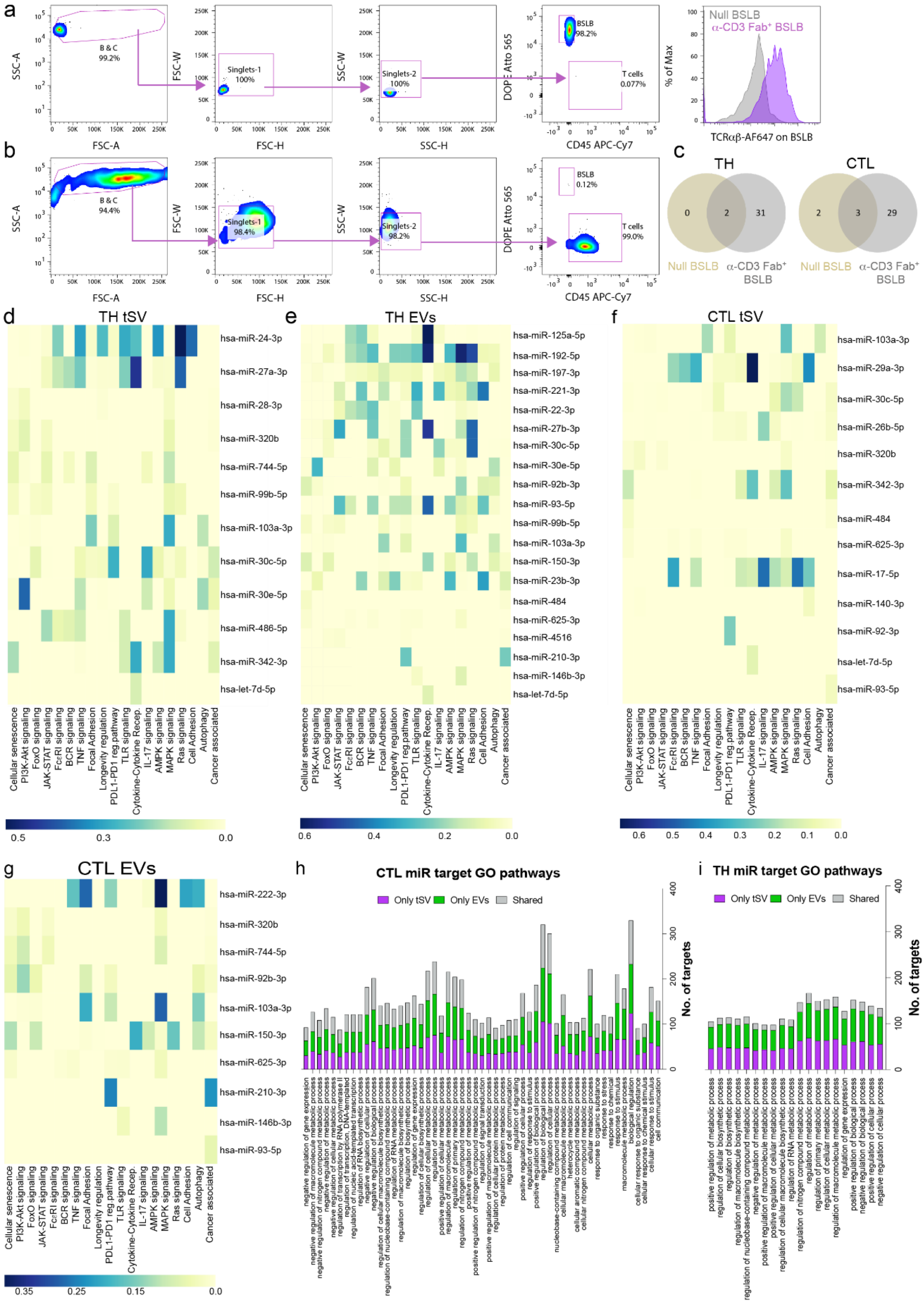
a
SSC-A
FSC-A
B & C
99.2%
FSC-W
FSC-H
Singlets-1
100%
SSC-W
SSC-H
Singlets-2
100%
DOPE Atto 565
CD45 APC-Cy7
BSLB
98.2%
T cells
0.077%
% of Max
Null BSLB
α-CD3 Fab⁺ BSLB
TCRαβ-AF647 on BSLB
b
B & C
94.4%
Singlets-1
98.4%
Singlets-2
98.2%
BSLB
0.12%
T cells
99.0%
c
TH
0
2
31
CTL
2
3
29
Null BSLB
α-CD3 Fab⁺ BSLB
d
TH tSV
hsa-miR-24-3p
hsa-miR-27a-3p
hsa-miR-28-3p
hsa-miR-320b
hsa-miR-744-5p
hsa-miR-99b-5p
hsa-miR-103a-3p
hsa-miR-30c-5p
hsa-miR-30e-5p
hsa-miR-486-5p
hsa-miR-342-3p
hsa-let-7d-5p
Cellular senescence
PI3K-Akt signaling
FoxO signaling
JAK-STAT signaling
FcεRI signaling
BCR signaling
TNF signaling
Focal Adhesion
Longevity regulation
PDL1-PD1 reg pathway
TLR signaling
Cytokine-Cytokine Recep.
IL-17 signaling
AMPK signaling
MAPK signaling
Ras signaling
Cell Adhesion
Autophagy
Cancer associated
0.5
0.3
0.0
e
TH EVs
hsa-miR-125a-5p
hsa-miR-192-5p
hsa-miR-197-3p
hsa-miR-221-3p
hsa-miR-22-3p
hsa-miR-27b-3p
hsa-miR-30c-5p
hsa-miR-30e-5p
hsa-miR-92b-3p
hsa-miR-93-5p
hsa-miR-99b-5p
hsa-miR-103a-3p
hsa-miR-150-3p
hsa-miR-23b-3p
hsa-miR-484
hsa-miR-625-3p
hsa-miR-4516
hsa-miR-210-3p
hsa-miR-146b-3p
hsa-let-7d-5p
0.6
0.4
0.2
0.0
f
CTL tSV
hsa-miR-103a-3p
hsa-miR-29a-3p
hsa-miR-30c-5p
hsa-miR-26b-5p
hsa-miR-320b
hsa-miR-342-3p
hsa-miR-484
hsa-miR-625-3p
hsa-miR-17-5p
hsa-miR-140-3p
hsa-miR-92-3p
hsa-let-7d-5p
hsa-miR-93-5p
0.6
0.5
0.4
0.3
0.2
0.1
0.0
g
CTL EVs
hsa-miR-222-3p
hsa-miR-320b
hsa-miR-744-5p
hsa-miR-92b-3p
hsa-miR-103a-3p
hsa-miR-150-3p
hsa-miR-625-3p
hsa-miR-210-3p
hsa-miR-146b-3p
hsa-miR-93-5p
0.35
0.25
0.15
0.0
h
CTL miR target GO pathways
Only tSV
Only EVs
Shared
No. of targets
i
TH miR target GO pathways
Only tSV
Only EVs
Shared
No. of targets

Supplementary Figure 9**. Pathway enrichment analyses reveals unique and shared miR in tSV and EVs sharing functional gene ontology categories**. **a** Sorting of BSLBs after conjugate dissociation for the sequencing of tSVs small RNA content. Shown is the gating strategy for the identification of single BSLB and cells in sorting experiments for the sequencing of small RNAs. *Right:* Shown are overlaid histograms of single BSLBs analyzed after sorting from T cell: BSLB co-cultures. Null (grey; containing only 20 molec./μm^2 of CD40 and 200 molec./μm^2 of ICAM1) and agonistic (violet; containing CD40, ICAM1 and 250 molec./μm^2 of anti-CD3ε^+ Fab) BSLBs are compared for the expression of T cell-derived TCR$\alpha\beta$. **b** Post-sort control showing the enrichment of single T cells by fluorescence-activated cell sorting as in (a). **c** Gene enrichment analyses for miR captured by null BSLBs and BSLBs containing 250 molec./μm^2 of anti-CD3ε Fab after coculturing with TH and CTL. **d-g** Heatmaps detailing miR species and KEGG GO pathways functionally enriched in TH tSV (**d**), TH EVs (**e**), CTL tSVs (**f**) and CTL EVs (**g**); Heatmap values represent log10 p-values. **h-i** Number of miR targets across different GO pathways. **h** CTL tSV and EVs miR targets are compared. **i** TH tSVs and EVs miR targets are compared. Shared miR targets per GO category are shown in grey, unique targets for tSVs are shown in magenta and unique targets for EVs miR are shown in green. Data from n = 8 biologically independent samples (donors; d-i) across six independent experiments. For functional and biological pathway enrichment analyses using MIENTURNET (d-i), P-values were determined by hypergeometric statistic test and FDR (Q = 0.05).

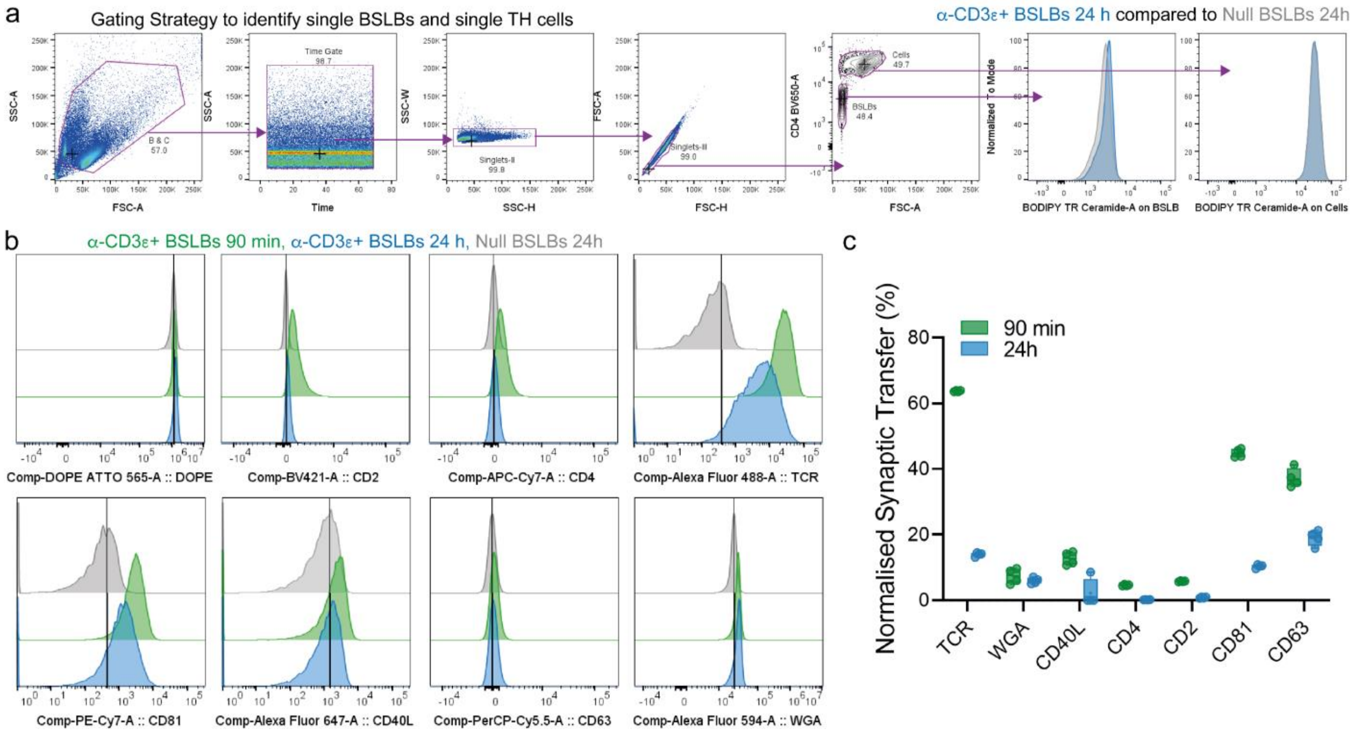

Supplementary Figure 10. **TSV release in BSLB: T cell co-cultures mimicking hours-long interactions between DC and T cells. a** Flow cytometry evaluation of T cell membrane transfer (BODIPY TR ceramide) to BSLBs after 24h incubation with TH cells. Shown is the gating strategy used to identify BSLBs and cells, and the side-by-side comparison of BODIPY TR Ceramide (membrane) transfer to agonistic BSLBs (α-CD3ε Fab^+, blue overlaid histograms) compared with null BSLBs (grey overlaid histograms, in left) and BODIPY TR expression in their respective cells (far-right overlaid histograms). **b-c** TSV capture by BSLB co-cultured with TH for either 90 min (green histograms) or 24 h (blue histograms) at 37°C and 5% CO_2. The fluorescence for DOPE ATTO 565 indicates that after 24 h of co-culture the supported lipid bilayers remain intact. **c** Normalized synaptic transfer (%) for the different markers shown in (**b**). In a-c, BSLBs were reconstituted with CD40 (500 molec./μm^2, reconstituting a DC membrane based on a measured density of 516 ± 216 molec./μm^2 on moDCs), ICAM1 (200 molec./μm^2) and α-CD3ε-Fab (250 molec./μm^2). Data representative of n = 2 (a) and 4 (b-c) biologically independent samples (donors) across two independent experiments.

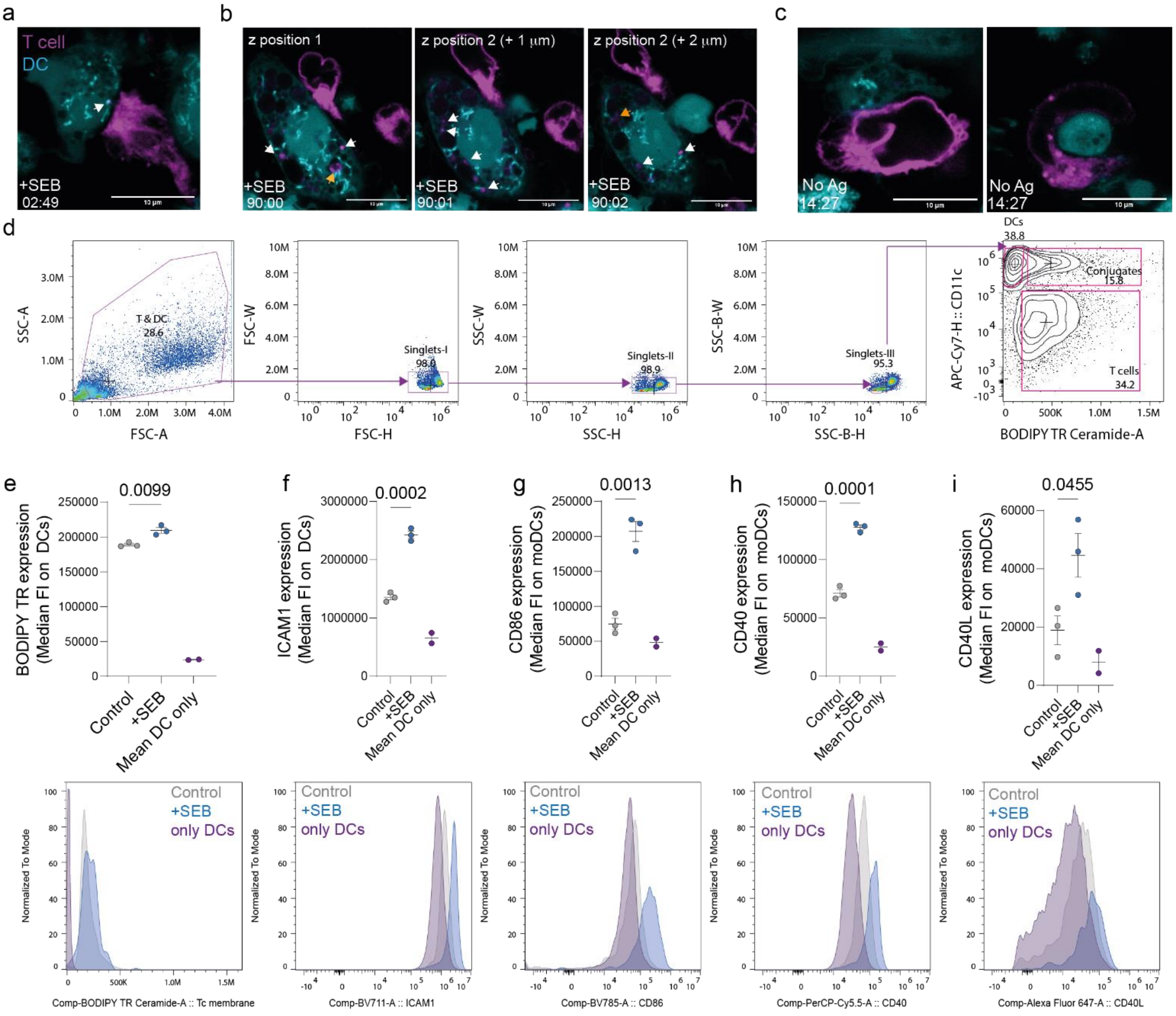

Supplementary Figure 11. **TH transfer membrane particles resembling tSV and activate antigen-presenting moDCs. a** Laser confocal microphotograph showing the transfer of membrane material from TH cells to antigen loaded monocyte-derived moDCs within 2 minutes of interaction. The image shows the transfer of diffraction-limited ceramide+ puncta (white arrow) from BODIPY TR ceramide (BTRC)-labelled TH to Staphylococcal Enterotoxin B (SEB; at 100 ng/mL[72])-loaded moDCs (cyan; labelled with Cell Trace Violet). **b** as in (a), laser confocal microphotograph showing the transfer of membrane material (white arrows) from TH cells to antigen loaded moDCs after 90 minutes of interaction and at three *z* planes. Note the polarization of TH cells, which resumed migration after 90 min of interaction. Orange arrows indicate the presence of large pieces of membrane reminiscent of material engulfed by trogocytosis[61]. **c** As in (b), moDCs devoid of superantigen (null) were cultured with TH cells. Most T cells failed to polarize their Golgi/ER and migrated by stablishing transient contacts with the DCs. **d** Flow cytometry gating strategy to identify single TH and single moDCs and measure membrane transfer (BTRC) between TH and moDCs after 24 h of co-culture. **e** Measurement of BTRC transfer from TH to moDCs either lacking (grey circles) or presenting SEB (blue circles) at 24 h of co-culture. **f-i** as in (d-e), moDC activation at 24 h of co-culture was measured by flow cytometry staining of ICAM1 (**f**), CD86 (**g**), CD40 (**h**), and CD40L (**i**)

on moDCs after conjugate dissociation using 2 mM EDTA in ice-cold PBS. The average value for moDCs cultured alone is shown for comparison (violet circles). *Bottom panels:* Representative overlaid histograms showing the fluorescence distribution of BTRC, ICAM1, CD86, CD40 and CD40L as shown in **e-i**. Histograms represent moDCs cultured alone (violet) or co-cultured with T cells in the absence (grey) or presence of SEB (blue). Data represent Means ± SEM of n = 2 (a-c) biologically independent samples across two independent experiments. In (e, d-i) 3 biologically independent samples across two independent experiments are shown. Statistical significance was determined by two-tailed, unpaired t-test (e-i). Values represent calculated P values for the indicated comparisons.

Supplementary Table 1. **List of antibodies used in this study.**

Antibody	Clone	Fluorochromes	Manufacturer	Dilution or concentration
anti-human CD2	TS1/8	Brilliant Violet 421™	BioLegend (San Diego, CA, #309217, Lot# B279601).	1:100 dilution.
anti-human CD4	A161A1	PerCP-Cy5.5	BioLegend (San Diego, CA, #357414, Lot# B316515).	1:100 dilution.
anti-human CD4	OKT4	PE-Cy7	BioLegend (San Diego, CA, #317414, Lot# B253231).	1:100 dilution.
anti-human CD4	OKT4	PE-Cy7	BioLegend (San Diego, CA, #317436, Lot# B284304).	1:200 dilution.
anti-human CD8α	HIT8a	Alexa Fluor® 700	BioLegend (San Diego, CA, #300920, Lot# B256905).	1:100 dilution.
anti-human CD11c	Bu15	APC-Cy7	BioLegend (San Diego, CA, #337218, Lot# B254813	1:800 dilution.
anti-human CD28	CD28.2	eFluor® 450	LifeTechnologies, eBioscience (#48-0289-42, Lot#).	1 μg/mL
anti-human CD28	CD28.2	Alexa Fluor® 700	BioLegend (San Diego, CA, #302920, Lot# non-available).	1 μg/mL
anti-human CD38	HB-7	Alexa Fluor® 700	BioLegend (San Diego, CA, #356624, Lot# B257486).	1:100 dilution.
anti-human CD39	A1	Brilliant Violet 510™	BioLegend (San Diego, CA, #328219, Lot# B303970).	1:100 dilution.
anti-human CD39	A1	Alexa Fluor® 647	BioLegend (San Diego, CA, #328202, Lot# B257126).	1 μg/mL
anti-human CD40	5C3	PerCP-Cy5.5	BioLegend (San Diego, CA, #334316, Lot# B256342).	1:200 dilution
anti-human CD40	G28.5	Alexa Fluor® 647*	BioLegend (#303602, Lot# B167433).	5 μg/mL
anti-human CD45	2D1	PE-Cy7	BioLegend (San Diego, CA, #368532, Lot# B285632).	1:200 dilution.
anti-human CD45	2D1	APC-Cy7	BioLegend (San Diego, CA, #368516, Lot# B289603).	1:100 dilution.
anti-human CD54	HA58	Brilliant Violet 711™	BD Horizon (#564078, Lot# B2032101).	1:200 dilution.
anti-human CD63	H5C6	PerCP-Cy5.5	BioLegend (San Diego, CA, #353020, Lot# B270203).	1:100 dilution.
anti-human CD73	AD2	Alexa Fluor® 647*	BioLegend (San Diego, CA, #344002, Lot# B216192).	1 μg/mL.
anti-human CD81	5A6	PE-Cy7	BioLegend (San Diego, CA, #349512, Lot# B276514).	1:100 dilution.
anti-human CD86	IT2.2	Brilliant Violet 785™	BioLegend (San Diego, CA, #305442, Lot# B337087).	1:200 dilution.
anti-human CD154 (CD40L)	24-31	Alexa Fluor® 488	BioLegend (San Diego, CA, #310815, Lot # B224765).	1 μg/mL for microscopy; 5 μg/mL for FCM.
anti-human CD154 (CD40L)	24-31	Alexa Fluor® 647	BioLegend (San Diego, CA, #310818, Lot# B266279).	1 μg/mL for microscopy; 5 μg/mL for FCM.
Anti-human CD156c (ADAM10)	SHM14	Alexa Fluor® 647*	BioLegend (#352702, Lot# B212788)	1 μg/mL
anti-human CD317 (BST2/Tetherin)	RS38E	Alexa Fluor® 647	BioLegend (San Diego, CA, #348404, Lot# B177299).	1 μg/mL
anti-human CD317 (BST2, PDCA-1)	26F8	Alexa Fluor® 488	eBioscience, Thermo Fisher Scientific Inc. (#53-3179-42, Lot# 2134438).	1 μg/mL
anti-human TCRαβ	IP26	Brilliant Violet 421™	BioLegend (San Diego, CA, #306722, Lot# B240856).	1 μg/mL
anti-human TCRαβ	IP26	Alexa Fluor® 488	BioLegend (San Diego, CA, #306712, Lot# B269884).	1 μg/mL
anti-human TCRαβ	IP26	Alexa Fluor® 647	BioLegend (San Diego, CA, #306714, Lot# B210817).	1 μg/mL
anti-human Perforin	B-D48	PE-Cy7	BioLegend (San Diego, CA, #353316, Lot# 353316).	5 μg/mL
Mouse IgG1 kappa Isotype control	X40	Brilliant Violet™ 421	BD Biosciences (#562438, Lot# 8242926)	1 μg/mL
Mouse IgG1 kappa Isotype control	P3.6.2.8.1	eFluor 450	BD Biosciences, #48-4714-82, Lot# B2191944).	1:100 dilution.
Mouse IgG1 kappa Isotype control	MOPC-21	Brilliant Violet 510™	BioLegend (San Diego, CA, #400172, Lot# B279185).	1 μg/mL
Mouse IgG1 kappa Isotype control	MOPC-21	Alexa Fluor® 488	BioLegend (San Diego, CA, #400129, Lot# B220820).	1 μg/mL
Mouse IgG1 kappa Isotype control	MOPC-21	Alexa Fluor® 647	BioLegend (San Diego, CA, #400130, Lot# B262021).	1 μg/mL
Mouse IgG1 kappa Isotype control	MOPC-21	Alexa Fluor® 700	BioLegend (San Diego, CA, #400144, Lot# B288791).	1 μg/mL
Mouse IgG1 kappa Isotype control	P3.6.2.8.1	Alexa Fluor® 647*	eBioscience, Thermo FIsher Scientific Inc. (MA, USA), #13-4714-85.	1 μg/mL
Mouse IgG2a kappa Isotype control	MOPC-173	Alexa Fluor® 647	BioLegend (San Diego, CA, #400240, Lot# B204560).	1 μg/mL
Mouse IgG1 kappa Isotype control	X40	Brilliant Violet™ 711	BD Biosciences (#563044, Lot# 0030270)	1 μg/mL
Mouse IgG2b kappa Isotype control	MPC-11	Brilliant Violet 785™	BioLegend (San Diego, CA, #400355, Lot# B228711).	1:100 dilution.
anti-human IgG Fc	Polyclonal	PE	Invitrogen (#12-4998-82, Lot# 1962116)	1:100 dilution.
Donkey anti-Rabbit IgG (H+L) minimal cross-reaction.	Polyclonal AffiniPure F(ab)2	Alexa Fluor 647	Jackson ImmunoResearch (#711-606-152, Lot# 119661)	1:2,000 dilution.
Rabbit anti-human TSG101	EPR7130B	Alexa Fluor 647	Abcam (#ab207664, Lot# GR260973-3)	1:100 dilution for microscopy.
Rabbit anti-human YB1	EPR22682-21	Unconjugated	Abcam (#ab255606, LOT# GR3285398-2)	1: 100 dilution for microscopy.
Rabbit anti-human SF3B3	EPR18440	Unconjugated	Abcam (#ab209402, LOT# GR255457-3)	1: 100 dilution for microscopy.
Rabbit Isotype control	EPR25A	Alexa Fluor 647	Abcam (#ab199093, LOT# GR317039-3)	1: 100 dilution for microscopy.
Rabbit anti-Vimentin	EPR3776	Alexa Fluor 555	Abcam (#ab203428, LOT# GR3281079-1)	1: 100 dilution for microscopy.
Rabbit anti-human CD40 Ligand	D5J9Y	Unconjugated	CST (#15094, Lot# 1)	1:500 dilution for immunoblotting.
Mouse anti-β-actin	8H10D10	Unconjugated	CST (#3700S, LOT# 18)	1:2,000 dilution for immunoblotting.
Rabbit anti-ALIX	EPR15314	Unconjugated	Abcam (#ab186429, LOT# GR3261765-6)	1:500 dilution for immunoblotting.
Rabbit anti-TSG101	EPR7130B	Unconjugated	Abcam (#ab125011, LOT#s GR299332-28)	1:500 dilution for immunoblotting.
Mouse anti-CD81	M38	Unconjugated	Invitrogen (#10630D, LOT# 01073933 and 00977863)	1:500 dilution for immunoblotting.
Donkey anti-Rabbit IgG	Non-reported	IRDye 800 CW	LI-COR (#926-32213, LOT# D01216-10)	1:15,000 dilution for immunoblotting.
Donkey anti-Mouse IgG	Non-reported	IRDye 680 RD	LI-COR (#926-68072, LOT# C91023-06)	1:15,000 dilution for immunoblotting.

Supplementary Table 2. **List of CRISPR/Cas9 gRNAs used in this study.**

Target	Alt-R CRISPR-Cas9 gRNA sequence (PAM underlined)	Catalog number (guide ID) of Alt-R CRISPR-Cas9
CD4	GAAGATGAGGGTGCACGAGTGGG	Hs.Cas9.CD4.1.AD
CD81	ATCTACATCCTCATCGCTGTGGG	Hs.Cas9.CD81.1.AB
ADAM10	CCCCATAAATACGGTCCTCAGGG	Hs.Cas9.ADAM10.1.AA
BST2	CCCCCAGAATCACGATGATCAGG	Hs.Cas9.BST2.1.AA
TSG101.AA	CTATCCGCCATACCAGGCAACGG	Hs.Cas9.TSG101.1.AA
TSG101.AB	GTGGCAGGATATGGACCACCAGG	Hs.Cas9.TSG101.1.AB
