# Supplementary material for: T-cell trans-synaptic vesicles are distinct and carry greater effector content than constitutive extracellular vesicles
Source: Nat Commun. 2022 Jun 16;13:3460. doi: 10.1038/s41467-022-31160-3 (PMC9203538; doi:10.1038/s41467-022-31160-3)
Supplement: Supplementary file 3 — Description of Additional Supplementary Files [file 41467_2022_31160_MOESM3_ESM.pdf]

Description of Additional Supplementary Files

Supplementary Data 1: List of inhibitors and concentrations used in this study.

Supplementary Data 2: EC50 for interferon-gamma release by different T cell clones used in this study.

Supplementary Data 3: miR enriched across all biologically independent samples (donors) analyzed in this study and separated by extracellular vesicle type.

Supplementary Data 4: List of targets identified for miR enriched, listed per extracellular vesicle type.
