# Supplementary material for: T-cell trans-synaptic vesicles are distinct and carry greater effector content than constitutive extracellular vesicles
Source: Nat Commun. 2022 Jun 16;13:3460. doi: 10.1038/s41467-022-31160-3 (PMC9203538; doi:10.1038/s41467-022-31160-3)
Supplement: Supplementary file 8 — Reporting Summary [file 41467_2022_31160_MOESM8_ESM.pdf]

nature portfolio

Corresponding author(s): Pablo F. Céspedes
Michael L. Dustin

Last updated by author(s): Feb 6, 2022

Reporting Summary

Nature Portfolio wishes to improve the reproducibility of the work that we publish. This form provides structure for consistency and transparency in reporting. For further information on Nature Portfolio policies, see our Editorial Policies and the Editorial Policy Checklist.

Statistics

For all statistical analyses, confirm that the following items are present in the figure legend, table legend, main text, or Methods section.

n/a	Confirmed	
☐	☒	The exact sample size (n) for each experimental group/condition, given as a discrete number and unit of measurement
☐	☒	A statement on whether measurements were taken from distinct samples or whether the same sample was measured repeatedly
☐	☒	The statistical test(s) used AND whether they are one- or two-sided<br>*Only common tests should be described solely by name; describe more complex techniques in the Methods section.*
☒	☐	A description of all covariates tested
☐	☒	A description of any assumptions or corrections, such as tests of normality and adjustment for multiple comparisons
☐	☒	A full description of the statistical parameters including central tendency (e.g. means) or other basic estimates (e.g. regression coefficient) AND variation (e.g. standard deviation) or associated estimates of uncertainty (e.g. confidence intervals)
☐	☒	For null hypothesis testing, the test statistic (e.g. F, t, r) with confidence intervals, effect sizes, degrees of freedom and P value noted<br>*Give P values as exact values whenever suitable.*
☒	☐	For Bayesian analysis, information on the choice of priors and Markov chain Monte Carlo settings
☒	☐	For hierarchical and complex designs, identification of the appropriate level for tests and full reporting of outcomes
☒	☐	Estimates of effect sizes (e.g. Cohen's d, Pearson's r), indicating how they were calculated

Our web collection on statistics for biologists contains articles on many of the points above.

Software and code

Policy information about availability of computer code

Data collection	Fortessa-X20, FACSCanto-II and AriaIII operating with FACSDiva v8.0,<br>Aurora Cytek operating with SpectroFlo v3.0,<br>Zeiss Airyscan 2 operating with Zen Blue v3.2,<br>TIRF-SIM instrument operating with LabVIEW v.16.0 and V-SIM v.1.7.00.0000.<br>NanoAnalyzer U30 instrument operating with nFCM Professional Suite v1.8,<br>Olympus TIRF Microscope operating with CellSense v1.15 build 14760,<br>Odyssey® CLx Near-Infrared detection system equipped with the Image Studio™ Lite v5.2 quantification software (LI-COR, Lincoln, NE) for immunoblot imaging.
Data analysis	Quality control and processing of the raw sequencing data were performed using sRNAbench 2019 release (https://bioinfo2.ugr.es/ceUGR/srnabench/) and miRQC (https://www.nature.com/articles/nmeth.3014#accession-codes).<br>Reads were aligned to the human genome reference (GRCh38.p13) using sRNAtoolboxDB.<br>MIENTURNET v 12.0.2 (http://userver.bio.uniroma1.it/apps/mienturnet/) was used for functional and biological pathway enrichment analyses and for miR mapping according to reference libraries (miRBase and MirGeneDB v2.0, and the KEGG (https://www.genome.jp/kegg/) database).<br>MEME (Multiple Em for Motif Elucidation 5.1.0) was used for motif analyses of bulk, enriched miR sequences in each sample were performed using The output of the 'miRs' module of miRNet 2.0 (https://www.mirnet.ca.) provided the motif miR-target gene interactome enrichment analysis.<br>Flow Cytometry analyses were performed with FlowJo LLC (v10.8.1).<br>TIRFM and confocal images were analysed using the ZEN 3.2 system blue edition v3.2 (Carl Zeiss Microscopy GmbH) and Fiji v2.1.0/1.53c (build 5f23140693).<br>Immunoblots were analyzed using Fiji v2.1.0/1.53c (build 5f23140693).

MaxQuant version 1.6.10.43, PANTHER v16.0 and Funset (http://funset.uno/) for LC-MS/MS analyses.
GraphPad Prism v8 for statistical analysis.

For manuscripts utilizing custom algorithms or software that are central to the research but not yet described in published literature, software must be made available to editors and reviewers. We strongly encourage code deposition in a community repository (e.g. GitHub). See the Nature Portfolio guidelines for submitting code & software for further information.

Data

Policy information about availability of data

All manuscripts must include a data availability statement. This statement should provide the following information, where applicable:
- Accession codes, unique identifiers, or web links for publicly available datasets
- A description of any restrictions on data availability
- For clinical datasets or third party data, please ensure that the statement adheres to our policy

All data-sets used for quantifications in this study are provided within the article, supplementary information and as source data. The mass spectrometry proteomics data generated in this study has been deposited to the ProteomeXchange Consortium via the PRIDE partner repository with the dataset identifier PXD033917 (https://www.ebi.ac.uk/pride/archive/projects/PXD033917). The RNA sequencing data generated in this study have been deposited in GEO under the accession number GSE181216 (https://www.ncbi.nlm.nih.gov/geo/query/acc.cgi?acc=GSE181216). The authors declare no restrictions on access to raw data, which will be available upon reasonable request from the corresponding authors.

Field-specific reporting

Please select the one below that is the best fit for your research. If you are not sure, read the appropriate sections before making your selection.

☒ Life sciences ☐ Behavioural & social sciences ☐ Ecological, evolutionary & environmental sciences

For a reference copy of the document with all sections, see nature.com/documents/nr-reporting-summary-flat.pdf

Life sciences study design

All studies must disclose on these points even when the disclosure is negative.

Sample size	No sample size calculations were performed in advance. In each case, we selected the sample size (n = number of biologically independent samples or donors) based on our previous experience with similar experiments.
Data exclusions	For experiments using CRISPR/Cas9-edited cells, those donors showing no down-regulation of the edited target were removed from analyses to properly address the question on whether the genetic ablation of T cell proteins affects the synaptic transfer of vesicles.
Replication	We performed at least two independent experiments including a minimum of three independent biological replicates (cells from independent donors).
Randomization	Samples derived from different donors, T cell types, drug treatments and CRISPR/Cas9-gRNAs were randomly allocated in our experimental plates and multi-well microscopy experiments.
Blinding	Single-blind acquisition and analyses were performed by our collaborators at NanoFCM. Size of extracellular vesicles are reported based on their analyses. Single-blind acquisition was also performed for the acquisition of drug and CRISPR/Cas9-synapse experiments by assigning unique identification numbers to each condition.

Reporting for specific materials, systems and methods

We require information from authors about some types of materials, experimental systems and methods used in many studies. Here, indicate whether each material, system or method listed is relevant to your study. If you are not sure if a list item applies to your research, read the appropriate section before selecting a response.

Materials & experimental systems

n/a	Involved in the study	
☐	☒	Antibodies
☒	☐	Eukaryotic cell lines
☒	☐	Palaeontology and archaeology
☒	☐	Animals and other organisms
☒	☐	Human research participants
☒	☐	Clinical data
☒	☐	Dual use research of concern

Methods

n/a	Involved in the study	
☒	☐	ChIP-seq
☐	☒	Flow cytometry
☒	☐	MRI-based neuroimaging

Antibodies

Antibodies used

anti-human CD2 clone TS1/8 Mouse IgG1, κ Brilliant Violet 421™ (BioLegend, #309217, LOT# B279601, B310536), used at 1:100 dilution.
anti-human CD4 clone A161A1 Rat IgG2b, κ PerCP-Cy5.5 (BioLegend, #357414, LOT# B316515), used at 1:100 dilution.
anti-human CD4 clone OKT4 Mouse IgG2b, κ PE-Cy7 (BioLegend, #317414, LOT# B253231), used at 1:100 dilution.
anti-human CD8α clone HIT8a Mouse IgG1, κ Alexa Fluor® 700 (BioLegend, #300920, LOT# B256905), used at 1:100 dilution.
anti-human CD28 clone CD28.2 Mouse IgG1, κ eFluor® 450 (LifeTechnologies, eBioscience, #48-0289-42) used at 1 µg/mL.
anti-human CD28 clone CD28.2 Mouse IgG1, κ Alexa Fluor® 700 (BioLegend, #302920, Lot# non-available) used at 1 µg/mL.
anti-human CD38 clone HB-7 Mouse IgG1, κ Alexa Fluor® 700 (BioLegend, #356624, LOT# B257486 and B303849), used at 1:100 dilution.
anti-human CD39 clone A1 Mouse IgG1, κ Brilliant Violet 510™ (BioLegend, #328219, LOT# B303970 and B240467), used at 1:100 dilution.
anti-human CD39 clone A1, Purified, conjugated in house with Alexa Fluor 647 (#328202, LOT# B257126), used at 1 µg/mL.
anti-human CD40 clone G28.5 Mouse IgG1, κ conjugated in house with Alexa Fluor® 647 (BioLegend, #303602, LOT# B167433), used at 5 µg/mL.
anti-human CD45 clone 2D1 Mouse IgG1, κ PE-Cy7 (BioLegend, #368532, LOT# B285632), used at 1:200 dilution.
anti-human CD45 clone 2D1 Mouse IgG1, κ APC-Cy7 (BioLegend, #368516, LOT# B289603), used at 1:100 dilution.
anti-human CD63 clone H5C6 Mouse IgG1, κ PerCP-Cy5.5 (BioLegend, #353020, LOT# B270203), used at 1:100 dilution.
anti-human CD73 clone AD2 Mouse IgG1, κ Purified antibody, conjugated in house with Alexa Fluor® 647 (BioLegend, #344002, LOT# B188932 and B216192), used at 1 µg/mL.
anti-human CD81 clone 5A6 Mouse IgG1, κ PE-Cy7 (BioLegend, #349512, LOT# B276514 and B295863), used at 1:100 dilution.
anti-human CD154 (CD40L) clone 24-31 Mouse IgG1, κ Alexa Fluor® 488 (BioLegend, #310815, LOT# B224765), used at 5 µg/mL.
anti-human CD154 (CD40L) clone 24-31 Mouse IgG1, κ Alexa Fluor® 647 (BioLegend, #310818, LOT# B266279, B309948), used at 5 µg/mL.
anti-human CD156c (ADAM10) clone SHM14 conjugated in house with Alexa Fluor 647 (BioLegend, #352702, LOT# B212788), used at 1 µg/mL.
anti-human CD317 (BST2/Tetherin) clone RS38E Mouse IgG1, κ Alexa Fluor® 647 (BioLegend, #348404, LOT# B177299), used at 1 µg/mL.
anti-human CD317 (BST2, PDCA-1) clone 26F8 Mouse IgG1, κ Alexa Fluor® 488 (eBioscience, Thermo Fisher Scientific Inc., #53-3179-42, LOT# 2134438), used at 1 µg/mL.
anti-human TCRαβ clone IP26 Mouse IgG1, κ Brilliant Violet 421™ BioLegend® (#306722, LOT# B240856), used at 1 µg/mL.
anti-human TCRαβ clone IP26 Mouse IgG1, κ Alexa Fluor® 488 BioLegend® (#306712, LOT# B269884, B312596), used at 1 µg/mL.
anti-human TCRαβ clone IP26 Mouse IgG1, κ Alexa Fluor® 647 BioLegend® (#306714, LOT# B210817), used at 1 µg/mL.
anti-human Perforin clone B-D48 Mouse IgG1, κ PE-Cy7 (BioLegend, #353316, LOT# B275993), used at 5 µg/mL.
anti-human CD40 PercPCy5.5 clone 5C3 (BioLegend, #334316, LOT# B256342),used 1: 200 dilution for spectral flow cytometry.
anti-human CD4 BV650 clone OKT4 (BioLegend, #317436, LOT# B284308 and B289735), used 1:200 dilution for spectral flow cytometry.
anti-human CD54 (ICAM1) BV711 clone HA58 (BD Horizon, #564078, LOT# B2032101 and #7143781), used at 1:200 dilution for spectral flow cytometry.
anti-CD11c APC-Cy7 clone Bu15 (BioLegend, #337218, LOT# B254813), used at 1:800 dilution for spectral flow cytometry.
anti-human CD86 BV785 clone IT2.2 (BioLegend, #305442, LOT# B286180, B337087). used at 1:200 dilution for spectral flow cytometry.
Mouse IgG1 kappa Isotype control clone X40 Brilliant Violet™ 421 (BD Biosciences, #562438, LOT# 8242926), used at 1 µg/mL.
Mouse IgG1 kappa Isotype control clone P3.6.2.8.1 eFluor 450 (BD Biosciences, #48-4714-82, LOT# 2191944), used at 1:100.
Mouse IgG1 kappa Isotype control clone MOPC-21 Alexa Fluor® 488 (BioLegend, #400129, LOT# B220820, B277964), used between 1 and 5 µg/mL depending on the effective concentration of the quantification antibody (e.g. anti-TCR or anti-CD154).
Mouse IgG1 kappa Isotype control clone MOPC-21 Brilliant Violet 510™ (BioLegend, #400172, LOT# B279185), used at 1 µg/mL.
Mouse IgG1 kappa Isotype control clone MOPC-21 Alexa Fluor® 647 (BioLegend, #400130, LOT# B262021), used between 1 and 5 µg/mL depending on the effective concentration of the quantification antibody (e.g. anti-TCR or anti-CD154).
Mouse IgG1 kappa Isotype control clone P3.6.2.8.1 Purified antibody conjugated in house with Alexa Fluor® 647 (eBioscience, Thermo FIsher Scientific Inc., #13-4714-85), used between 1 and 5 µg/mL depending on the effective concentration of the quantification antibody (e.g. anti-TCR or anti-CD154).
Mouse IgG2a kappa Isotype control clone MOPC-173 Alexa Fluor 647 (BioLegend, #400240, LOT# B204560), used between 1 and 5 µg/mL depending on the effective concentration of the quantification antibody.
Mouse IgG1 kappa Isotype control clone MOPC-21 Alexa Fluor® 700 (BioLegend, #400144, LOT# B288791), used at 1 µg/mL.
Mouse IgG1 kappa Isotype control clone X40 Brilliant Violet 711 (BD Biosciences, #563044, LOT# 0030270), used at 1 µg/mL.
Mouse IgG2b kappa Isotype control clone MPC-11 Brilliant Violet 785 (BioLegend, #400355, LOT# B228711), used at 1:100 dilution.
anti-human IgG Fc, polyclonal, PE (Invitrogen, #12-4998-82, Lot# 196116), used at 1:100 dilution.
Rabbit anti-human TSG101 clone EPR7130B AF647 (#ab207664, LOT# GR260973-3), used at 1:100 dilution for microscopy.
Rabbit anti-human YB1 (YBX1) clone EPR22682-21 (Abcam, #ab255606, LOT# GR3285398-2 and GR3285398-4), used at 1:100 dilution for microscopy.
Rabbit anti-human SF3B3 clone EPR18440 (Abcam, #ab209402, LOT# GR255457-3), used at 1:100 dilution for microscopy.
Rabbit Isotype control clone EPR25A AF647 (Abcam, #ab199093, LOT# GR317039-3), used at 1:100 dilution for microscopy.
Rabbit anti-Vimentin clone EPR3776 AF555 (Abcam, #ab203428, LOT# GR3281079-1), used at 1:100 dilution for microscopy.
Rabbit anti-human CD40 Ligand clone D5J9Y (CST, #15094), used at 1:500 dilution for immunoblotting (western blotting).
Mouse anti-β-actin clone 8H10D10 (CST, #3700S, LOT# 18), used at 1:2,000 dilution for immunoblotting (western blotting).
Rabbit anti-TSG101 clone EPR7130B (Abcam, #ab125011, LOT#s GR299332-28 and GR299332-29), used at 1:500 dilution for immunoblotting (western blotting).

	Rabbit anti-ALIX clone EPR15314 (Abcam, #ab186429, LOT# GR3261765-6). Used at 1:500 dilution for immunoblotting.<br>Mouse anti-CD81 clone M38 (Invitrogen, #10630D, LOT# 01073933 and 00977863). Used at 1:500 dilution for immunoblotting.<br>IRDye 800 CW Donkey anti-Rabbit IgG (LI-COR, #926-32213, LOT# D01216-10). Used at 1:15,000 dilution for immunoblotting.<br>IRDye 680 RD Donkey anti-Mouse IgG (LI-COR, #926-68072, LOT# C91023-06). Used at 1:15,000 dilution for immunoblotting.
Validation	Antibodies were titrated to determine optimal concentrations for staining and quantifications. All antibodies have been previously validated in the literature and in previous work using supported lipid bilayers (Saliba et al. 2021: https://elifesciences.org/articles/47528). The specificity of our stainings was controlled by the use of Knock-out primary cells, irrelevant cells and the use of antibody isotype controls conjugated with the same fluorochrome. In some experiments, due to reduced sample volumes, we mixed two monoclonal primary antibodies in immunoblots (TSG101 and ALIX). We performed independent immunoblots with TSG101 and ALIX to demonstrate the identification of discrete bands separated by around 50 kDa, and consistent with the predicted size of each protein.

Flow Cytometry

Plots

Confirm that:

- [x] The axis labels state the marker and fluorochrome used (e.g. CD4-FITC).
- [x] The axis scales are clearly visible. Include numbers along axes only for bottom left plot of group (a 'group' is an analysis of identical markers).
- [x] All plots are contour plots with outliers or pseudocolor plots.
- [x] A numerical value for number of cells or percentage (with statistics) is provided.

Methodology

Sample preparation	Prior to experiments dead cells were removed using either Ficoll-Paque or Lymphoprep 1,077 g/mL density centrifugation. Cells were either washed twice to remove culture media, or centrifuged to remove assay media (HBS/HSA), then cells were cooled down gradually 15 min at RT and a minimum of 40 min on ice. Cells were then resuspended in staining antibody master mix containing previously defined optimal antibody concentrations in ice-cold 5% BSA-PBS, mixed gently and incubated for a minimum of 30 min on ice. Cells were then washed twice with PBS pH7.4 and resuspended in 100 uL of PBS pH7.4 for acquisition using the High throughput samplers of BD LSR Fortessa-X20, BD FACSCanto II or Cytek Aurora.<br><br>For NanoFCM experiments, The concentration of samples was determined by comparison to 250 nm silica nanoparticles of known concentration to calibrate the sample flow rate. EV isolates were sized according to standard operating procedures using a proprietary 4-modal silica nanosphere cocktail (NanoFCM Inc., S16M-Exo). Using the NanoFCM software (NanoFCM Profession V1.8), a standard curve was generated based on the side scattering intensity of the four different silica particle populations of 68, 91, 113 and 155nm in diameter. Silica provides a stable and monodisperse standard with a refractive index of approximately 1.43 to 1.46, which is close to the range of refractive indices reported in the literature for EVs (n = 1.37 to 1.42). The laser was set to 10 mW and 10% SSC decay.
Instrument	BD LSR Fortessa X20, BD FACSCanto II, LSR Fortessa-X20, NanoAnalyzer U30 instrument (NanoFCM Inc.), Cytek Aurora.
Software	FlowJo V9 to v10.
Cell population abundance	Sorted samples were acquired to control for residual contamination. As shown in Supplementary Fig. 7a, sorted BSLBs and cells were used in downstream analyses if the purity was >99%. A minimum of 1,000 events were recorded to analyse post-sort purity.
Gating strategy	For most experiments we used FSC-A/SSC-A for identifying cells and cells and BSLBs, followed by FSC-W/FSC-H, then SSC-W/SSC-H, and FSC-A/FSC-H to exclude doublets. For most samples we performed a QC step to check the continuity of events over acquisition time (FSC-W versus FSC-H, followed by SSC-W versus SSC-H) and on those rare cases where a time gap was observed an additional gate of time continuum was selected to ensure measurements of events free of optical aberrations.

- [x] Tick this box to confirm that a figure exemplifying the gating strategy is provided in the Supplementary Information.
